# Supplementary material for: Splice-Junction-Based Mapping of Alternative Isoforms in the Human Proteome
Source: Cell Rep. Author manuscript; Available in PMC 2020 Jan 15. (PMC6961840; doi:10.1016/j.celrep.2019.11.026)
Supplement: 2 [file NIHMS1546469-supplement-2.zip › DF1/PXD010154/Testis/Testis_10_CLTCL1_LQEHFQLQNLGINPANIGFSTLTMESDK.pdf]

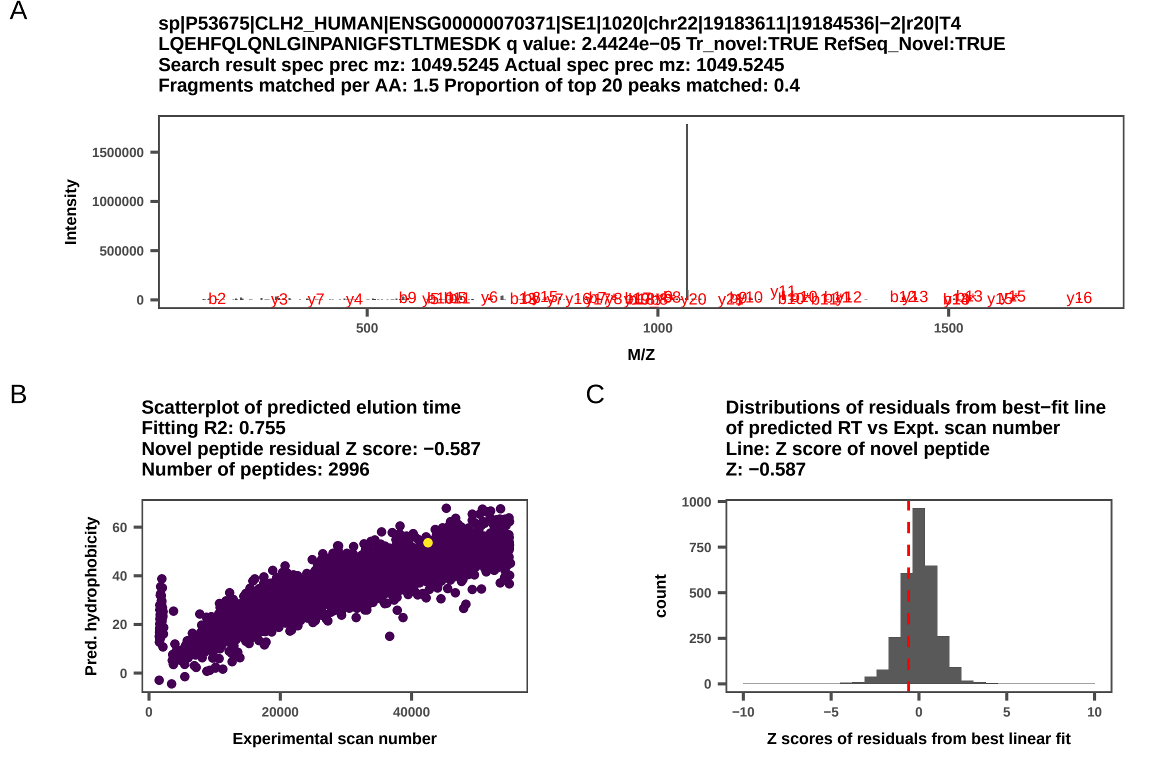
A
sp|P53675|CLH2_HUMAN|ENSG00000070371|SE1|1020|chr22|19183611|19184536|−2|r20|T4
LQEHFQLQNLGINPANIGFSTLTMESDK q value: 2.4424e−05 Tr_novel:TRUE RefSeq_Novel:TRUE
Search result spec prec mz: 1049.5245 Actual spec prec mz: 1049.5245
Fragments matched per AA: 1.5 Proportion of top 20 peaks matched: 0.4
Intensity
1500000
1000000
500000
0
b2
y3
y7
y4
b9
y6
y20
y11
y16
500
1000
1500
M/Z
B
Scatterplot of predicted elution time
Fitting R2: 0.755
Novel peptide residual Z score: −0.587
Number of peptides: 2996
Pred. hydrophobicity
60
40
20
0
0
20000
40000
Experimental scan number
C
Distributions of residuals from best−fit line
of predicted RT vs Expt. scan number
Line: Z score of novel peptide
Z: −0.587
count
1000
750
500
250
0
−10
−5
0
5
10
Z scores of residuals from best linear fit
